# Experimental Infection and the Effects of Temperature on the Pathogenicity of the Infectious Spleen and Kidney Necrosis Virus in Juvenile Nile Tilapia (*Oreochromis niloticus*)

**DOI:** 10.3390/ani14030452

**Published:** 2024-01-30

**Authors:** Tarcísio Martins França e Silva, Guilherme Alves de Queiróz, Carlos Augusto Gomes Leal

**Affiliations:** Department of Preventive Veterinary Medicine, Veterinary School, Federal University of Minas Gerais, Belo Horizonte 31270-901, Brazil; tarcisiomfes@gmail.com (T.M.F.e.S.); guiaqua7@yahoo.com.br (G.A.d.Q.)

**Keywords:** control, fish farming, heat treatment, iridovirus, ISKNV, qPCR

## Abstract

**Simple Summary:**

In this study, we examined how different water temperatures affect the severity of illness caused by the infectious spleen and kidney necrosis virus in juvenile tilapia and also analyzed the amount of the virus that remains in the fish at different temperatures. We found that the best conditions occurred at higher temperatures, specifically at 32 and 34 °C, where the fish showed no mortality. Furthermore, specific tests for the virus revealed a significant reduction in the viral load in fish tissues. On the other hand, when the fish were kept at lower temperatures, such as 26, 28, and 30 °C, they developed the disease and some died, indicating that the virus was more active in these conditions. These results are consistent with previous studies on aquatic animals. For example, research on shrimp showed that the white spot virus infection was inhibited at 31 °C, and carps infected with the herpes virus were found to be immune after being treated at 30 °C. Based on these results, we propose the use of preventive heat treatment against the infectious spleen and kidney necrosis virus in juvenile tilapia, taking into consideration similar findings in other aquatic animal species to control viral diseases.

**Abstract:**

The infectious spleen and kidney necrosis virus (ISKNV) is one of the most important emerging viral pathogens for Nile tilapia (*Oreochromis niloticus*) farming. While prevalent worldwide, it has recently been detected in Brazil. However, despite the importance of the virus and the affected fish species, there are no scientific data on the effects of water temperature on disease pathogenesis in Nile tilapia. In the present study, we conducted two trials using juvenile Nile tilapia over a 15-day period. In trial 1, an experimental infection model was developed based on the intraperitoneal inoculation of active viral homogenates (4.3 × 10^4^ virus fish^−1^), while control fish were similarly inoculated with inactivated viral homogenates. In trial 2, the fish were maintained at different water temperatures (26, 28, 30, 32, and 34 °C) and then infected with ISKNV. For virus detection, kidney and spleen samples were collected and analyzed by qPCR. Our results show that the disease was successfully reproduced in experimental conditions with active homogenates, with the first signs of the disease appearing on the third day after infection. In addition, a significant reduction in mortality was observed in the groups maintained at higher temperatures (>30 °C). This suggests that a treatment of the disease with non-lethal hyperthermia can be used to control the symptoms and mortality of ISKNV-infected Nile tilapia juveniles.

## 1. Introduction

The rapid growth in global aquaculture production has contributed significantly to nutritional development and food security worldwide. The cultivation of Nile tilapia (*Oreochromis niloticus*) has posed as one of the most important and promising sectors of global aquaculture, with an estimated yield of 4.5 million tons in 2018, with Brazil ranking as the fourth largest producer in the world [1]. However, high stocking densities and frequent management, which are necessary in intensive production systems, reduce fish immunity and favor the maintenance and spread of pathogens. With the increasing production of tilapia worldwide, the emergence of infectious diseases that have the potential to cause serious damage to the sector poses as a constant concern for producers [2].

The infectious spleen and kidney necrosis virus (ISKNV) is a large double-stranded DNA-containing icosahedral virus belonging to the genus *Megalocytivirus* of the family *Iridoviridae* [3]. It is a globally distributed virus that causes serious economic losses to the aquaculture industry. Associated with high-mortality outbreaks in several species of marine and freshwater fish, ISKNV has emerged as one of the main viral agents in tilapia cultures on various continents [4]. The initial detection of the virus infecting tilapia was in 2015 in Thailand [5], followed by outbreaks in the USA in 2016 [4] and in Ghana in 2019, whereby estimated mortality rates between 60% and 90% were observed [6]. The disease was first detected in Brazil in 2020 [7].

Strategies to prevent and control viral diseases in fish through fluctuations in water temperature have been investigated among several cultivated species as the temperature of the culture water directly affects the ability of the virus to infect host cells [8,9]. Despite the importance of the results obtained in investigations on the latter among several commercially cultivated aquatic species, there are still no scientific data on the effects of temperature on the pathogenesis of infections caused by ISKNV in tilapia. Thus, the main objectives of this study were to standardize the first model of the experimental infection of juvenile tilapia by ISKNV and to evaluate the effects of water temperature on the pathogenesis of viral infections. Based on the literature data, we hypothesized that heat treatment can inhibit the virus’s multiplication and can potentiate the clearance of this disease in Nile tilapia.

## 2. Materials and Methods

### 2.1. Sample Collection

To obtain viral samples, juvenile tilapia exhibiting clinical signs suggestive of ISKNV infection were collected during outbreaks from farms with a positive history of this disease. These animals were submitted to the Routine Bacteriology Laboratory (Veterinary School, Federal University of Minas Gerais, Belo Horizonte, Brazil) for diagnosis through specific qPCR testing for ISKNV. The sampled fish were also subjected to bacteriological and parasitological examination. Kidney and spleen samples from fish positive for ISKNV and negative for other pathogens were collected aseptically and stored at −80 °C until use to prepare the viral homogenates for experimental infection.

### 2.2. Molecular Detection: DNA Extraction and qPCR

The total DNA from approximately 20 mg of the organ pool (spleen + kidney) from field-collected fish and from fish that died in the experimental infection trials was extracted using the Wizard DNA Extraction kit (Promega, Madison, WI, USA), following the manufacturer’s instructions. The qPCR was performed according to the MIQE guidelines [10] to detect and quantify ISKNV, as previously described by [11], with modifications. For the reactions, the GoTaq^®^ Probe Master Mix kit (Promega, Madison, WI, USA) was used, yielding a final volume of 25 µL, containing 900 nm of each primer and 600 nm of the probe. The qPCR conditions were as follows: an initial step of 95 °C for 20 s, 40 cycles at 95 °C for 1 s, and a final step of 60 °C for 20 s. Forward (5′-TGA CCA GCG AGT TCC TTG ACT T-3′), reverse (5′-CAT AGT CTG ACC GTT GGT GAT ACC-3′), and RSIV probe primers that were doubly labeled with fluorophore (FAM) at the 5′ end and quencher (BHQ-1) at the 3′ end (FAM-AAC GCC TGC ATG CCT GGC-BHQ-1) were used. Samples were evaluated in triplicate and were considered positive when the average number of quantification cycles was <35. The QuantStudio 1 platform (Thermo Scientific, Waltham, MA, USA) was used in the qPCR analyses. The samples were evaluated in 96-well plates (Thermo Scientific, Waltham, MA, USA). In each run, a DNA sample of the RSIV strain KagYT-96, provided by the OIE reference lab, was used as a positive control and ultrapure water as a negative control.

### 2.3. Preparation of Viral Homogenates

The viral homogenates used in the infection experiments were prepared according to Go and Whittington [12,13], with modifications. A pool was made composed of approximately 0.1 g of the kidney and spleen from positive animals that were collected in the field. The material was placed in 1.5 mL microtubes containing 900 µL of a minimal essential medium (MEM, Sigma-Aldrich, St. Louis, MO, USA) at a 1:9 ratio. Tissues were macerated using a sterile pestle until complete disruption was achieved. The tissue suspensions were then homogenized in a vortex mixer for 30 s and centrifuged at 1000 rpm for 10 min. The supernatant was then transferred to a new tube and diluted 1:1 in MEM. The total volume was doubly filtered through 0.45 µm and 0.22 µm membranes (Jetbiofil, Guangzhou, China). For the inoculation of the control groups, half of the total volume of the filtered viral homogenate was inactivated at 65 °C for one hour in a thermoblock. The volume of the active and inactive samples was then stored at −80 °C until use. Two hours before the infection, the homogenates were warmed to 4 °C and homogenized in a vortex mixer. An active viral homogenate measuring 100 µL was also separated for total DNA extraction, followed by the detection and quantification of the viral load by qPCR. The viral load was determined by qPCR using a standard curve with a DNA sample of the red sea bream iridovirus (RSIV) strain KagYT-96 of known concentrations provided by the OIE reference laboratory for the RSIV disease at the National Research Institute of Aquaculture, Japan.

### 2.4. Experimental Design

Two trials were performed using juvenile Nile tilapia with an average weight of 5 ± 1 g, acquired from a commercial fish farm. The animals were housed in 57 L glass aquariums with continuous water flow (renewal rate of 2 L h^−1^) and constant supplemental aeration at temperatures of 26 °C for trial 1 and, according to the experimental group for trial 2, ranging from 26 °C to 34 °C. The animals were fed a commercial ration containing 45% crude protein (CP) in the proportion of 2% of the live weight day^−1^, twice a day. At the time of experimental infection, the animals were anesthetized in a bath of 100 mg L^−1^ of a benzocaine solution (Sigma-Aldrich, USA). After infection, the animals were observed daily to assess clinical signs. Dead animals were collected over a period of 15 days. In the two trials, 10 fish per group were used without aquarium replicates. Brazilian laws for Animal Welfare and Ethics in Scientific Research recommends avoiding the use of replicates if the statistical power of the study is sufficient to support the results obtained. Based on the number of fish used in the groups (*n* = 20 for fingerlings in trial 1; and n = 10 in trial 2), a minimum effect of 60% and 75% (mortality in challenge groups) in trials 1 and 2, respectively, as well as an acceptable type I error of 5%, our experiment shows a power of the statistical tests that is higher than 80% (calculated by the software G*Power version 3.1.9.6 [14]), which is considered good or recommended for biological studies. All procedures performed with animals in the present study were approved by the Ethics Committee on Animal Use of the Federal University of Minas Gerais (protocol number 101/2021).

In both trials, the cumulative mortality was calculated as the percentage of animals that died during the experimental period in relation to the initial number of fish challenged according to the following formula: cumulative mortality = 100 × (dead fish in each treatment during experimental period/number of fish challenged per treatment).

#### 2.4.1. Trial 1: Development of the Experimental Infection Model

In this trial, 44 fish were used, four of which were randomly sampled to perform a specific qPCR assay for ISKNV to confirm the absence of the pathogen. The remaining animals were separated into four groups, one group per aquarium and 20 fish per group. The animals were acclimatized for 10 days in 57 L aquariums under the same rearing conditions as mentioned above.

The animals in the virus-exposed groups were inoculated intraperitoneally (IP) with 50 µL of active viral homogenates (4.3 × 10^4^ virus fish^−1^; the viral load was calculated using the standard curve method with DNA isolated from a known quantity of the red sea bream iridovirus (RSIV) strain KagYT-96 provided by the OIE). The fish in the control group were inoculated with 50 µL of the thermally inactivated viral homogenates. During this experimental period, kidney and spleen samples were collected from dead animals for ISKNV analysis by qPCR. At the end of the experimental period, all surviving animals were euthanized with an overdose of benzocaine (300 mg L^−1^), and kidney and spleen samples were collected for ISKNV-specific qPCR.

#### 2.4.2. Trial 2: Effect of Temperature on the Pathogenicity of ISKNV

A total of 110 fish were used in this study. The animals were acclimatized for 10 days in a 120 L aquarium under the same rearing conditions as mentioned above. Ten fish were randomly collected for molecular testing to confirm the absence of pathogens (mainly ISKNV).

After confirming the absence of pathogens, individuals were homogeneously divided into ten 57 L aquariums (10 fish per aquarium), and the temperatures were adjusted according to the different experimental groups, as described in Table 1.

For experimental infection, active and inactivated viral homogenates were prepared as described in Section 2.3. The challenged groups were inoculated with 50 µL of the filtered homogenate (4.3 × 10^4^ virus fish^−1^), while the control groups were inoculated with 50 µL of the heat-inactivated homogenate. The dead fish were immediately subjected to necropsy to remove the kidney and spleen. Samples were fractionated, and a portion was stored at −80 °C for subsequent DNA extraction, detection, and quantification by qPCR. The remaining portion was fixed in a 10% buffered formalin for a histological analysis.

### 2.5. Histopathology

For the histopathological analyses, cross-sections of the spleen and kidney tissues were collected from all experimental fish and fixed in a 10% buffered formalin for 48 h before preservation in 70% ethanol. Subsequently, 2–3 mm fragments were embedded in a histological paraffin, cut on a Leica RM-2245 rotary microtome (Leica Biosystems, Nussloch, Germany) to a thickness of 3–4 µm, mounted on glass slides, stained with hematoxylin and eosin (H&E) according to standard protocols for histology [15], and examined under a light microscope. Samples containing lesions characteristic of hypertrophied cells in the presence of intracytoplasmic bodies in one or more tissues were considered positive.

### 2.6. Statistical Analysis

The final mortality rates between the control and infected groups were compared using the Chi-square test, whereby *p* < 0.05 was considered statistically significant. To evaluate the effects of temperature on mortality, the relative risk reduction (RRR) was calculated using the Mantel–Haenszel method, with a confidence interval of 95%. The analyses were performed using the GraphPad Prism Version 7.05 software (GraphPad Software Inc., San Diego, CA, USA).

## 3. Results

### 3.1. Experimental Infection Model

**Figure 1:**
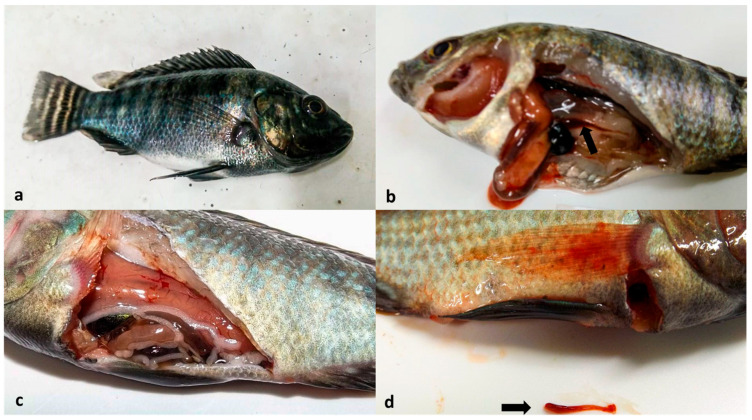
Moribund animals from the infected group showing melanosis (**a**), gill pallor (**b**), visceral pallor (**c**), and spleen hypoplasia (arrows in (**b**,**d**)).

The first clinical signs of the disease, such as apathy, lethargy (animals at the bottom of the aquarium), anorexia, and melanosis (Figure 1a), were observed on the third day post-infection. The diseased fish showed other clinical signs such as ascites and exophthalmos. During necropsy, pale viscera, exudate in the coelomic cavity, gill pallor, and splenic hypoplasia were observed (Figure 1b–d). By contrast, in the control group, no clinical signs of disease or mortality were observed during the experimental period. At the end of the experiment, all negative control animals tested negative for ISKNV infection.

**Figure 2:**
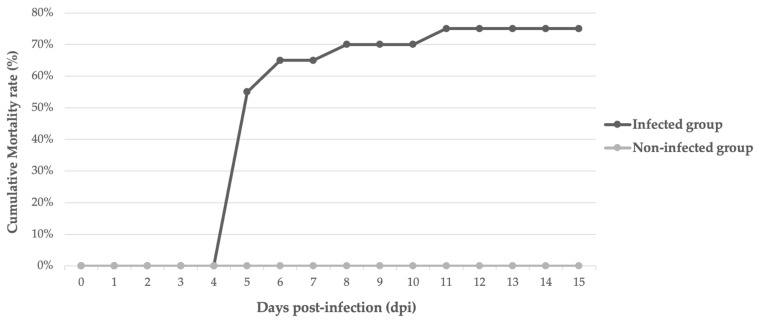
Cumulative mortalities in juveniles of tilapia housed in a water temperature of 26 °C during the period of experimental ISKNV infection.

Daily mortality rates during the experimental period are shown in Figure 2. The first mortality was recorded on the fifth day post-infection and marked the peak of daily mortality, with 11 dead fish (55%). Two animals died on day six (65%), one on day eight (70%), and one on day eleven (75%). All animals in the group infected with active homogenates showed positive results for the virus in the qPCR analysis for ISKNV.

### 3.2. Effects of Temperature on ISKNV Virulence

Based on trial 2, a clear influence of temperature on the pathogenicity of the virus was observed in the challenged groups. None of the control groups kept at different temperatures and inoculated with the heat-inactivated homogenate showed mortality or clinical signs of ISKNV or other infectious diseases throughout the experimental period, and at the end, all animals tested negative for ISKNV. In the infected group of fish maintained at 26 °C, six dead fish (60%) were recorded, all of which tested positive for ISKNV. Of the four surviving animals in this group, two tested positive (carriers) and two tested negative. In the infected group kept at 28 °C, four dead fish (40%) were recorded, all of which showed positive results from the qPCR test. Of the six surviving fish in this group, only one tested positive (carrier) and five tested negative for the virus. In the group of infected fish maintained at 30 °C, three dead animals (30%) were recorded, all of which tested positive for the virus. Of the seven surviving fish in this group, two tested positive for the virus (carriers) and five tested negative. In the infected group maintained at 32 °C, there was no record of mortality, and all fish at the end of the period tested negative for the virus. Similarly, there was no record of mortality in the infected group maintained at 34 °C. However, two surviving fish tested positive for ISKNV. Table 2 shows the relative risk (RR), relative risk reduction (RRR), 95% confidence interval, and Chi-square test results for the infected groups compared to the 26 °C group, which was considered a positive control in the present study.

### 3.3. Histopathology

**Figure 3:**
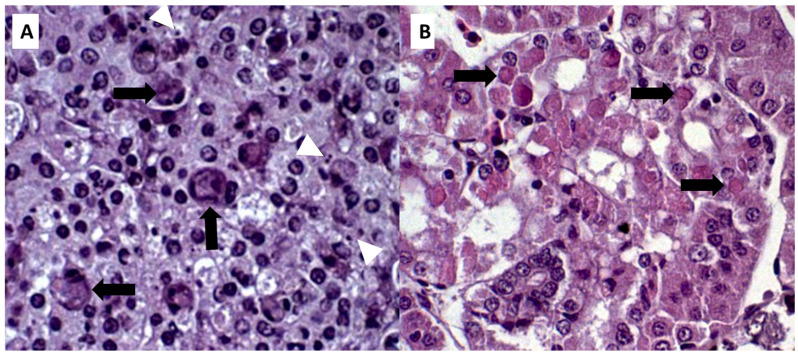
Histological sections of the kidney of Nile tilapia (*Oreochromis niloticus*) experimentally infected with ISKNV. Megalocytes in the spleen can be seen with the presence of intracytoplasmic basophilic bodies (arrows) and apoptotic figures (white arrowheads). H&E, 60× magnification (**A**); Histological fragment showing a region of renal cortex, represented by renal tubules with the presence of hyaline degeneration and increased cytoplasmic volume, with the presence of intracytoplasmic eosinophilic corpuscles. H&E, 60× magnification (**B**).

A histopathological examination of the spleen sourced from diseased animals revealed a predominance of white pulp, composed mainly of lymphoid cells and macrophages, with hyaline degeneration. In some lymphoid cells, an enlargement of the cytoplasm (megalocytes) was observed, with the presence of intracytoplasmic basophilic bodies (suggestive of viral particles) that repelled the nucleus to the periphery (Figure 3A). In addition, apoptotic figures were detected in the lymphocytes. A histopathological examination of kidney fragments revealed areas of predominantly hyaline tubular degeneration, with intracytoplasmic bodies completely filling the cytoplasm of the tubular epithelial cells (Figure 3B). In conjunction, it was possible to identify the discrete presence of hydropic degenerated apoptotic figures in the renal tubules and a mesangial cell response in rare renal glomeruli.

## 4. Discussion

Although ISKNV was reported to affect marine fish in Asia as early as the 1990s, the evolution of the virus in recent years has led to an increase in the range of susceptible species, as well as the geographic distribution of the virus. Since the outbreaks of ISKNV in farmed tilapia reported in Thailand in 2015 [5], the disease has quickly emerged in other countries such as Ghana [6], the United States [4], and lastly Brazil [7], demonstrating the importance of controlling this problem. The use of experimental infection models is essential for a better understanding of the disease dynamics of emerging fish diseases and thus for the development of control strategies. Nevertheless, obtaining ISKNV for experimental infections is not an easy task since isolation of the virus from freshwater fish is laborious and difficult. Therefore, the main alternative method is by the inoculation of viral homogenates from the tissues of naturally infected fish. The present study was successful in proposing an experimental infection model for ISKNV using juvenile Nile tilapia that were IP injected with viral homogenates obtained from diseased tilapia. Go et al. [12] reported similar results in terms of clinical symptoms and mortality after the experimental injection of ISKNV homogenates from dwarf gourami (*Colisa lalia*) in Murray cod (*Maccullochella peelii*) fingerlings. With the experimental infection standardized, future studies of viral pathogenesis, control, and treatment can be carried out to better understand and deal with the disease in Nile tilapia.

The treatment of viral diseases in fish by temperature manipulation has been reported in the literature. Exposure of koi carp (*Cyprinus carpio koi*) and common carp (*Cyprinus carpio*) to the koi herpes virus (KHV) at permissive temperatures followed by a change to higher temperatures (30 °C) has been used successfully in fish destined for export to Israel [8]. These temperature treatments significantly reduce mortality, and fish that survive the process are marketed as “immune to the disease” [16]. Similar results and benefits were also reported by You et al. [17] after high water temperatures (27 and 31 °C) significantly reduced mortality in Japanese shrimp (*Marsupenaeus japonicus*) experimentally infected with white spot syndrome virus (WSSV). In our study, the effects of different water temperatures on ISKNV pathogenicity were evaluated, as well as on the remaining viral load in infected fish submitted to the different water temperatures. The best results were obtained at the two highest temperatures (32 and 34 °C) to which the fish were exposed, with no mortalities. In addition, the ISKNV qPCR tests performed on day 15 post-infection showed a significant reduction in viral DNA in the tissues of these animals. On the other hand, the fish kept at the lowest temperatures (26, 28, and 30 °C) developed the disease and suffered mortality, evidencing the infectivity of the virus in this temperature range. These results are similar to those observed in other aquatic animals, such as the shrimp virus. These experiments showed an inhibition of WSSV infectivity in shrimp as well as a reduction in the viral load at 31 °C [17]. Also, another study evaluating carps infected with KHV classified those animals that survived the temperature treatment at 30 °C as “immune” [16]. The proposal for the use of preventive heat treatment against ISKNV in juvenile tilapia takes into account the results of studies conducted with other species of farmed aquatic animals to control viral diseases, as in, for example, the prevention of WSSV infection in penaeid shrimp, where heat treatment is already a routine practice in the field [18].

Fish farming in enclosed greenhouses is already a common practice, especially in larvicultures during the winter, to ensure optimal temperature for fish reproduction and offspring rearing. In this sense, greenhouses can be adapted and used by tilapia farms for the reception of juveniles and thus provide a prophylactic treatment against the virus. These data support the possibility of applying heat treatment for the prevention of ISKNV in tilapia farming in Brazil, since ideal thermal conditions for treatment efficacy are easily achieved, as the country is naturally favored by a hot climate. Future studies should be conducted to verify whether selective hyperthermic treatments and for specific periods of time are able to produce the same effect in fish.

## 5. Conclusions

The infection model successfully reproduced the disease under experimental conditions, and an increasing water temperature promoted a significant reduction in the pathogenicity of ISKNV in juvenile tilapia. These results corroborate several studies already published on the importance of the culture water temperature for the manifestation and development of viral infections in fish. Furthermore, they point to the possibility of using hyperthermic treatment in the field as a control and prevention tool against ISKNV in tilapia cultures.

## Figures and Tables

**Table 1:** Experimental groups, viral challenge, and the different water temperatures tested.

Experimental Group	*n*	Challenge with ISKNV	Water Temperature
G1-I	10	Viral homogenate	26 °C
G1-C	10	Inactivated viral homogenate	26 °C
G2-I	10	Viral homogenate	28 °C
G2-C	10	Inactivated viral homogenate	28 °C
G3-I	10	Viral homogenate	30 °C
G3-C	10	Inactivated viral homogenate	30 °C
G4-I	10	Viral homogenate	32 °C
G4-C	10	Inactivated viral homogenate	32 °C
G5-I	10	Viral homogenate	34 °C
G5-C	10	Inactivated viral homogenate	34 °C

**Table 2:** Mortality and statistical analysis of experimental groups subjected to different water temperatures.

Experimental Group	Mortality								Survivors		
	Yes		No								
	*n*	%	*n*	%	RR ^1^	RRR % ^2^	CI_95%_^3^	*p* ^4^	Total	qPCR +	qPCR −
G1-I (26 °C)	6	60	4	40	-	-	-	-	4	2	2
G2-I (28 °C)	4	40	6	60	0.67	33.33	0.328–0.337	0.0046	6	1	5
G3-I (30 °C)	3	30	7	70	0.50	50	0.492–0.507	0.00002	7	2	5
G4-I (32 °C)	0	0	10	100	0	100	-	>0.00001	10	0	10
G5-I (34 °C)	0	0	10	100	0	100	-	>0.00001	10	2	8

^1^ relative risk. ^2^ relative risk reduction. ^3^ confidence interval of 95%. ^4^ value *p*: Chi-square test.

## Data Availability

Data are contained within the article.
